# Supplementary material for: Causal Relationship between Aging and Anorexia Nervosa: A White-Matter-Microstructure-Mediated Mendelian Randomization Analysis
Source: Biomedicines. 2024 Aug 16;12(8):1874. doi: 10.3390/biomedicines12081874 (PMC11351342; doi:10.3390/biomedicines12081874)
Supplement: Supplementary file 1 [file biomedicines-12-01874-s001.zip › Supplementary Figure/Figure S8/Leaveoneout plot of MR analysis for TL to ubm-b-1787.pdf]

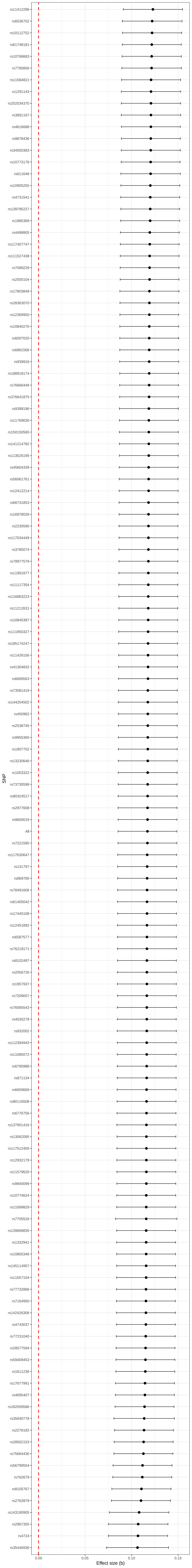
rs11412296
rs6536702
rs10112752
rs61748181
rs10768683
rs7790856
rs11584821
rs1291143
rs202034370
rs3891167
rs4616688
rs9878436
rs34550383
rs10773176
rs611646
rs10905255
rs4731541
rs139795227
rs1985369
rs4498805
rs117407747
rs111527438
rs7099229
rs2555104
rs17803849
rs28363070
rs12369950
rs10840270
rs6007020
rs6881568
rs939916
rs188918174
rs76666449
rs376641875
rs9398196
rs11769630
rs150150565
rs141214782
rs113525195
rs45604339
rs56061761
rs12412214
rs66731853
rs16978028
rs2230590
rs117034449
rs3785074
rs79977579
rs11991877
rs11117354
rs116863223
rs11212631
rs10845387
rs111950327
rs185174247
rs11426156
rs41304832
rs6669563
rs73581419
rs144204502
rs450962
rs2538745
rs9955360
rs1907702
rs13230646
rs1003322
rs73730598
rs80324517
rs2977608
rs9600019
All
rs7221585
rs117630647
rs131797
rs869785
rs78491606
rs61405042
rs17445108
rs12451892
rs6587577
rs76219171
rs8102497
rs2056726
rs1957937
rs7209057
rs76065543
rs4530278
rs932002
rs112394943
rs11085072
rs6790988
rs871134
rs6659669
rs80116508
rs6776756
rs137901416
rs13062095
rs117512405
rs12932179
rs11579626
rs9940099
rs10774624
rs11699829
rs7705526
rs139669835
rs1332941
rs10805346
rs145114957
rs11557154
rs77732866
rs7164950
rs142426306
rs4743037
rs77231040
rs28577594
rs59409453
rs1611236
rs17677991
rs4695407
rs182059586
rs35640778
rs2276182
rs28502153
rs75664430
rs56799554
rs762679
rs8105767
rs2763979
rs143190905
rs2967355
rs4724
rs35446936
SNP
0.00
0.05
0.10
0.15
Effect size (b)
